# Parasitoid-mediated horizontal transmission of *Rickettsia* between whiteflies

**DOI:** 10.3389/fcimb.2022.1077494

**Published:** 2023-01-04

**Authors:** Yuan Liu, Zi-Qi He, Qin Wen, Jing Peng, Yu-Tong Zhou, Nasser Mandour, Cindy L. McKenzie, Muhammad Z. Ahmed, Bao-Li Qiu

**Affiliations:** ^1^ Chongqing Key Laboratory of Vector Insects, College of Life Sciences, Chongqing Normal University, Chongqing, China; ^2^ Guangdong Laboratory for Lingnan Modern Agriculture, Guangzhou, China; ^3^ Key Laboratory of Bio-Pesticide Innovation and Application of Guangdong Province, South China Agricultural University, Guangzhou, China; ^4^ Department of Plant Protection, Faculty of Agriculture, Suez Canal University, Ismailia, Egypt; ^5^ Subtropical Insects and Horticulture Research Unit, Agricultural Research Service, Unite States Department of Agriculture (USDA), Fort Pierce, FL, United States

**Keywords:** Bemisia tabaci, endosymbionts, Rickettsia, Encarsia formosa, horizontal transmission

## Abstract

Intracellular bacterial endosymbionts of arthropods are mainly transmitted vertically from mother to offspring, but phylogenetically distant insect hosts often harbor identical endosymbionts, indicating that horizontal transmission from one species to another occurs in nature. Here, we investigated the parasitoid *Encarsia formosa*-mediated horizontal transmission of the endosymbiont *Rickettsia* between different populations of whitefly *Bemisia tabaci* MEAM1. *Rickettsia* was successfully transmitted from the positive MEAM1 nymphs (*R* ^+^) into *E. formosa* and retained at least for 48 h in *E. formosa* adults. Fluorescence *in situ* hybridization (FISH) visualization results revealed that the ovipositors, mouthparts, and digestive tract of parasitoid adults get contaminated with *Rickettsia*. Random non-lethal probing of *Rickettisia-*negative (*R^−^*) MEAM1 nymphs by these *Rickettsia-*carrying *E. formosa* resulted in newly infected MEAM1 nymphs, and the vertical transmission of *Rickettsia* within the recipient females can remain at least up to F3 generation. Further phylogenetic analyses revealed that *Rickettsia* had high fidelity during the horizontal transmission in whiteflies and parasitoids. Our findings may help to explain why *Rickettsia* bacteria are so abundant in arthropods and suggest that, in some insect species that shared the same parasitoids, *Rickettsia* may be maintained in populations by horizontal transmission.

## 1 Introduction

Intracellular bacteria often live with innumerable species of arthropods including insects. Interaction between bacteria and their hosts may be parasitic, symbiotic, or neutral (Bourtzis and Miller, 2006). These endosymbionts can broadly be divided into primary (obligate) endosymbionts and secondary (facultative) endosymbionts (Bing et al., 2013a; Bing et al., 2013b; Gnankine et al., 2013). The primary endosymbionts (such as *Portiera aleyrodidarum* in whiteflies) can supply essential nutrients under limited or unbalanced hosts’ diets thus essential for host survival (Douglas, 1998; Baumann, 2005). Secondary endosymbionts on the other hand are not essential for host survival; however, recent studies have revealed that they play other important roles in ecological and evolutionary phenomena, such as manipulating reproduction of their hosts (Segoli et al., 2013; Baldini et al., 2014; Staudacher et al., 2017; Thongprem et al., 2020; Wang et al., 2020; Candasamy et al., 2022; Li et al., 2022). Normally, many arthropod individuals harbor more than one species of endosymbionts, and they are mainly inherited vertically from mother to offspring with high fidelity, which is higher than 99.0% homology between the generations (Karut et al., 2020; Shan et al., 2020). However, their intra- or interspecifically horizontal transmission can also occur (Chiel et al., 2009; Oliver et al., 2010; Li et al., 2017a; Liu et al., 2020b).

The routes and mechanisms driving horizontal transmission of bacterial endosymbionts have been studied extensively in the last two decades (Woodbury et al., 2013; Zhang et al., 2013; Ilinsky et al., 2022). Several studies reported parasitoid-mediated horizontal transmission among phylogenetically distant species (Duron et al., 2010; Gehrer and Vorburger, 2012; Ahmed et al., 2015). For instance, *Arsenophonus*-uninfected parasitoids can acquire endosymbiont infection while developing inside an infected host (Duron et al., 2010). Similarly, the parasitoid *Eretmocerus furuhashii* may also serve as a phoretic vector, spreading *Wolbachia* from positive whitefly *Bemisia tabaci* AsiaII7 to its negative populations (Ahmed et al., 2015). *Hamiltonella defensa* and *Regiella insecticola* endosymbionts can be efficiently transmitted when a parasitoid sequentially stabs an infected and then an uninfected aphid (Gehrer and Vorburger, 2012). Here, we report, for the first time, the efficient phoretic transfer of *Rickettsia* from infected to uninfected members of whitefly *B. tabaci* MEAM1 cryptic species (Middle East Asia Minor 1), by one of the dominant parasitoid species, *Encarsia formosa*.

The whitefly *B. tabaci* (Gennadius) (Hemiptera: Aleyrodidae) is a small sucking agricultural pest that consists of more than 30 morphologically indistinguishable cryptic species (Dinsdale et al., 2010; Hu et al., 2011; Lee et al., 2013). This whitefly pest can cause severe economic losses due to its ability to directly suck phloem sap from plants, indirectly secrete honeydew leading to sooty mold development, and, most importantly, transmit numerous plant viruses (Jones, 2003; Cuthbertson and Vänninen, 2015). *B. tabaci* harbors various bacterial endosymbionts, including *Arsenophonus*, *Cardinium*, *Hamiltonella*, *Rickettsia*, and *Wolbachia*, and among them (Lv et al., 2021), *Rickettsia* could modify host biology and manipulate the host’s reproduction to enhance its spread (Gottlieb et al., 2006; Moran et al., 2008; Shi et al., 2021). Recent reports revealed two localized distribution patterns of *Rickettsia* inside the whitefly body, “scattered” and “confined” (Gottlieb et al., 2008; Shi et al., 2018), with scattered patterns that might be contributing to the horizontal transmission of *Rickettsia* (Chiel et al., 2009; Caspi-Fluger et al., 2012; Shi et al., 2016). To date, biological control using parasitoids plays a vital role in the sustainable control of insect pests, including whitefly. *E. formosa* Gahan (Hymenoptera: Aphelinidae) is one of the most commonly used parasitoids in commercial whitefly control worldwide. It is a primary, solitary, thelytokous endoparasitoid that oviposits right inside the nymph of its host but penetrates whitefly nymphs with its ovipositor and mouth parts to examine them first before feeding or laying eggs (Lenteren et al., 1996; Gerling et al., 2001; Bacci et al., 2010; Li et al., 2011; Sugiyama et al., 2011).

In the current study, we used polymerase chain reaction (PCR), quantitative real-time PCR (qPCR), and fluorescence *in situ* hybridization (FISH) to demonstrate the intraspecific horizontal transmission of *Rickettsia* from infected to uninfected whitefly individuals through parasitoid *E. formosa.* We anticipate that *Rickettsia*, because of horizontal transmission, could modify the biology of the transinfected host individual and might enhance its pestiferous nature.

## 2 Materials and methods

### 2.1 Whiteflies and parasitoids

Two populations of *B. tabaci* (MEAM1 cryptic species) were used for the study: *Rickettsia*-positive (*R* ^+^) and *Rickettsia*-negative (*R^−^*) (Liu et al., 2020a). Both populations were then reared for at least 10 generations on cotton plants (*Gossypium hirsutum* L. var. Lumianyan no. 32) under standard laboratory conditions of 26 ± 2°C, 60% RH, and a photoperiod of 14:10 (L:D) h. Meanwhile, in order to ensure the purity of the two populations during experiments, approximately 100 adult whiteflies were selected to check the biotype and presence/absence of *Rickettsia* every month (Qiu et al., 2009; Shi et al., 2018).

Parasitoid *E. formosa* was initially collected from parasitized *B. tabaci* on tomato plants (*Lycopersicon esculentum* Mill.) in the Institute of Beijing Academy of Agriculture and Forestry Sciences. The previous studies have revealed that this *E. formosa* strain is stably infected with *Wolbachia* (Fan, 2013). For further experimental study, two separate parasitoid cultures were established, one with *Rickettsia*-positive MEAM1 nymphs and the other with *Rickettsia*-negative MEAM1 nymphs fed on cotton plants. Both parasitoid cultures were then maintained on the different host species for many generations under ambient conditions [26 ± 2°C, 60% RH, and a 14:10 (L/D) h photoperiod].

### 2.2 PCR detection of endosymbionts in parasitoids

Approximately 90 parasitoid adults that emerged from *Rickettsia*-negative MEAM1 nymphs were randomly selected for the testing of endosymbionts (*Wolbachia*, *Rickettsia*, *Cardinium*, *Hamiltonella*, *Hemipteriphilus*, *Arsenophonus*, *Fritschea*, and *Portiera*). Total DNA was extracted from a single parasitoid following the method of Ahmed et al. (2010), which tested 90 individuals. All PCR reactions were run in a 25-μl buffer containing 1 μl of the template DNA lysate, 1 μl of each primer, 2.5 mM MgCl_2_, 200 mM for each dNTP, and 1 unit of DNA Taq polymerase (Invitrogen, Guangzhou, China) (Shi et al., 2018). The genus-specific primers used in this study are shown in **Table 1**. The remainder of amplified PCR products were then sent to the Beijing Institute (BGI) for sequencing after expected bands were visible on a 1.5% agarose gel containing Gold-View colorant. In order to confirm the specificity of the detection, the DNA of the endosymbiont *Wolbachia* was used as the positive control, and ddH_2_O was used as the negative control.

**Table 1:** Details of primers used in this study.

Target gene	Primer sequence (5′-3′)	Reference
*Wolbachia*	Forward: 5’-TGGTCCAATAAGTGATGAAGAAAC-3’	Zhou et al., 1998
(*wsp*)	Reverse: 5’-AAAAATTAAACGCTACTCCA-3’	
*Rickettsia*	Forward: 5’-GCTCAGAACGAACGCTATC-3’	Gottlieb et al., 2006
(*16S rRNA*)	Reverse: 5’-GAAGGAAAGCATCTCTGC-3’	
(*gltA*)	Forward: 5’-TCCTATGGCTATTATGCTTG-3’	Caspi-Fluger et al., 2012
	Reverse: 5’-CCTACTGTTCTTGCTGTGG-3’	
(*Pgt*)	Forward 1: 5’-AGGTTTAGGCTAGTCTACACG-3’	Caspi-Fluger et al., 2012
	Reverse 1: 5’-GTCTACGCACGATTGATG-3’	
	Forward 2: 5’-ACTCATGAAATTATCGGCACAG-3’	
	Reverse 2: 5’-GCATGAATTTGGCACTTAAGC-3’	
*Cardinium*	Forward: 5’-GCGGTGTAAAATGAGCGTG-3’	Weeks et al., 2003
(*16S rRNA*)	Reverse: 5’-ACCTMTTCTTAACTCAAGCCT-3’	
*Hamiltonella*	Forward: 5’-TGAGTAAAGTCTGGAATCTGG-3’	Chiel et al., 2007
(*16S rRNA*)	Reverse: 5’- AGTTCAAGACCGCAACCTC-3’	
*Hemipteriphilus*	Forward: 5’-GCTCAGAACGAACGCTRKC-3’	Bing et al., 2013a
(*16S rRNA*)	Reverse: 5’-TTCGCCACTGGTGTTCCTC-3’	
*Arsenophonus*	Forward: 5’-CGTTTGATGAATTCATAGTCAAA-3’	Thao and Baumann, 2004
(*16S rRNA*)	Reverse: 5’- GGTCCTCCAGTTAGTGTTACCCAAC-3’	
*Fritschea*	Forward: 5’-GATGCCTTGGCATTGATAGGCGATGAAGGA-3’	Everett et al., 2005
(*16S rRNA*)	Reverse: 5’-TGGCTCATCATGCAAAAGGCA-3’	
*Portiera*	Forward: 5’-TGCAAGTCGAGCGGCATCAT-3’	Zchori-Fein and Brown, 2002
(*16S rRNA*)	Reverse: 5’-AAAGTTCCCGCCTTATGCGT-3’	
*Rickettsia*	Forward: 5’-CGGATTGCTTTACTTAC-3’	Pan et al., 2013
(*q-gltA*)	Reverse: 5’-AAATACGCCACCTCTA-3’	
*B. tabaci*	Forward: 5’-TCTTCCAGCCATCCTTCTTG-3’	Ghanim and Kontsedalov, 2009
(*β-actin*)	Reverse: 5’-CGGTGATTTCCTTCTGCATT-3’	
*E. formosa*	Forward: 5’-CGCCACGAGACCGATAGC-3’	Fan, 2013
(*28S rRNA*)	Reverse: 5’-GTAAGCCAAAGAGGTTGACGATG-3’	

### 2.3 *Rickettsia* transmission from *R* ^+^ whiteflies to parasitoids

To study whether *Rickettsia* could be infected in the parasitoid *E. formosa*, 30 newly emerged parasitoid adults were collected from the *R* ^+^ MEAM1 nymphs while the other 30 newly emerged parasitoids were collected from the *R^−^* MEAM1 hosts (this was treated as one experimental replicate), and three replicates were performed. They were examined for the presence of *Rickettsia* using the *Rickettsia*-specific primers listed in **Table 1** (*16S rRNA*, *gltA*, and *Pgt*) (Gottlieb et al., 2006; Caspi-Fluger et al., 2012). The total DNA samples were extracted using a TIANamp Genomic DNA kit (Tiangen, Beijing, China), whereas the diagnostic PCR, and the sequencing of DNA fragments were performed with essentially the same methods as described above. The PCR detection included positive (*Wolbachia*) and negative (ddH_2_O) controls.

### 2.4 Quantitative and FISH detection of *Rickettsia* in parasitoids

qPCR was used to detect the relative titers of *Rickettsia* in the different tissues of *E. formosa*, and the *28S rRNA* gene of *E. formosa* was used as the housekeeping gene. The *q-gltA* gene of *Rickettsia* and the *28S rRNA* gene of *E. formosa* qPCR detection are shown in **Table 1**. Approximately 30 newly emerged parasitoid adults from *R* ^+^ MEAM1 nymphs were dissected under the stereomicroscope, including head, thorax, and abdomen, and divided into three repeats for testing. Amplifications were performed using Thunderbird SYBR Green PCR mix (TOYOBO, Osaka, Japan). The cycling conditions were as follows: 5 min activation at 95°C, 40 cycles of 30 s at 95°C, 30 s at 55°C, and finally 30 s at 72°C (Ghanim and Kontsedalov, 2009). A non-template negative control was included for each primer set to check for primer dimers and contamination.

To further determine the *Rickettsia* localization in *E. formosa*, parasitoid adults (age, 5–7 days) that developed from the *R* ^+^ MEAM1 nymphs were randomly selected and put in Carnoy’s fixative (chloroform:ethanol:acetic acid = 6:3:1) for FISH. FISH detections were performed with the *Rickettsia*-specific *16S rRNA* gene probe (Rb1-Cy3:5’-Cy3-TCCACGTCGCCGTCTTGC-3’), as the method described by Sakurai et al. (2005) and Gottlieb et al. (2006). Following this, stained parasitoid samples were mounted and observed under a Nikon eclipse Ti-U inverted microscope. The specificity of detection was confirmed using a no-probe control.

### 2.5 Persistence of *Rickettsia* in parasitoids

In order to monitor the persistence of *Rickettsia* in *E. formosa*, newly emerged parasitoid adults (considered F1 generation) that were collected from the *R* ^+^ MEAM1 nymphs were put into a 5 cm × 1.5 cm (length × diameter) glass tube sealed with gauze. The parasitoids were then fed on 20% honey water using filter paper at 0, 8, 16, 24, 32, 40, and 48 h, respectively; 30 individuals were ground together in each replicate for qPCR; and each stage qPCR detection was repeated three times. Afterwards, all parasitoid adults were captured again and stored into a refrigerator at −80°C, and the total DNA samples were extracted using a TIANamp Genomic DNA kit (Tiangen, Beijing, China). The 28*S rRNA* gene of *E. Formosa* was used as the housekeeping gene, whereas *Rickettsia* qPCR detection was performed with essentially the same methods, as described previously.

### 2.6 *Rickettsia* transmission from parasitoids to *R^−^* whiteflies and its vertical transmission in whiteflies

In the laboratory, approximately 50 pairs of *R^−^* MEAM1 adults were released into a separate leaf cage (6 cm diameter × 4 cm height) to reproduce on clean cotton leaves for 24 h, with clips subsequently fixed to them to prevent whiteflies from escaping. When the progeny from these adults developed to third instar nymphs, which is the stage preferred by *E. formosa* parasitoids, 160 nymphs were randomly selected and the remaining ones were removed. Afterwards, eight parasitoid adults (age, 2–3 days) that developed from *R* ^+^ MEAM1 nymphs were introduced into the cages to probe and feed on these third instar *R^−^* MEAM1 nymphs for 8 h, and the probing behavior of the parasitoids was observed under a stereomicroscope, which was treated as one replicate and each experiment included 20 parallel replicates (20 × 160 = 3,200 whitefly nymphs). When the survivor *R^−^* MEAM1 nymphs developed to adults (the average proportion of such samples was 9.85 ± 0.47%), 30 newly emerged adults were collected into a 1.5-ml Eppendorf tube for DNA extraction and *Rickettsia* PCR detection. The DNA of endosymbiont *Portiera* was used as a positive control and ddH_2_O was used as a negative control to eliminate possible confounding variables, and the experiment was repeated three times.

Another group of newly emerged whitefly adults, which developed from the nymphs of *Rickettsia*-carrying parasitoids that were non-lethally probed or fed on, was used to determine whether *Rickettsia* was vertically transmitted between whitefly generations. About 20 pairs of these whitefly adults were introduced into a leaf cage containing cotton leaves and were given 24 h to mate and oviposit before removal, and the newly emerged F1 adults were used to produce the F2 generation and then the F3 generation. The *Rickettsia* PCR detection procedure was the same as above. The *β-actin* gene of *B. tabaci* was used as the housekeeping gene (Ghanim and Kontsedalov, 2009); the experiment was repeated three times.

### 2.7 Phylogenetic analysis of *Rickettsia* in whiteflies and parasitoids

The homology of *Rickettsia* endosymbionts in positive donor MEAM1, negative recipient MEAM1, and the parasitoid vector *E. formosa* was phylogenetically analyzed. The *16S rRNA*, *gltA*, and *pgt* gene sequences are listed in **Table 2** and were edited and aligned using Lasergene v7.1 (DNASTAR, Inc., Madison, WI). Two phylogenetic trees of *Rickettsia* were conducted based on the *16S RNA* and *gltA* genes; meanwhile, eight *16S rRNA* and eight *gltA* sequences of *Rickettsia* from different insect hosts were selected as reference for homologous analysis in the GenBank database using basic local alignment search tools. Bayesian information criterion was used to select the best model and partitioning scheme in PartitionFinder v. 1.0.1 (Lanfear et al., 2012). Finally, phylogenetic trees of *Rickettsia* were generated by IQ-TREE v1.6.8 using the TIM3e+I and K3Pu+F+R2 model based on the maximum likelihood (ML) method with 1,000 non-parametric bootstrap replications in RAxML (Stamatakis, 2006).

**Table 2:** The reference sequences of *Rickettsia* used in phylogenetic analysis.

Groups	Hosts	GenBank accession numbers		
		*16S rRNA*	*gltA*	*Pgt*
Transitional	*Aulogymnus trilineatus*	FJ609405	FJ666769	NA
	*Aulogymnus balani/skianeuros*	FJ609406	FJ666770	NA
Adalia	*Subcoccinella vigintiquattuorpunctata*	FJ609398	FJ666762	NA
	*Adalia bipunctata*	FJ609400	FJ666765	NA
Bellii	*Bemisia tabaci*	DQ077707	DQ077708	JN940922
Melloidae	*Meloidae* sp.	FJ609389	FJ666754	NA
Rhizobius	*Rhizobius litura*	FJ609388	FJ666753	NA
Outgroup	*Orientia tsutsugamushi*	NR025860	NA	NA
	*Citrate synthase*	NA	U59716	NA

NA, not available.

### 2.8 Statistical analyses

A Bio-Rad machine (American) and the accompanying software (Bio Rad CFX Manager) were used for qPCR data normalization, and the relative titers of *Rickettsia* in different treatments were calculated using the method of 2^−^ΔΔct. All dates, such as acquisition and persistence of *Rickettsia* in parasitoids, and vertical transmission of *Rickettsia* in recipient *R^−^* whiteflies, were analyzed using one-way analysis of variance (ANOVA), and means were compared using the Duncan’s test (SPSS 17.0) at *p* < 0.01. All figures were drawn with Sigmaplot 10.0.

## 3 Results

### 3.1 Detection of the endosymbionts in parasitoids

**Figure 1:**
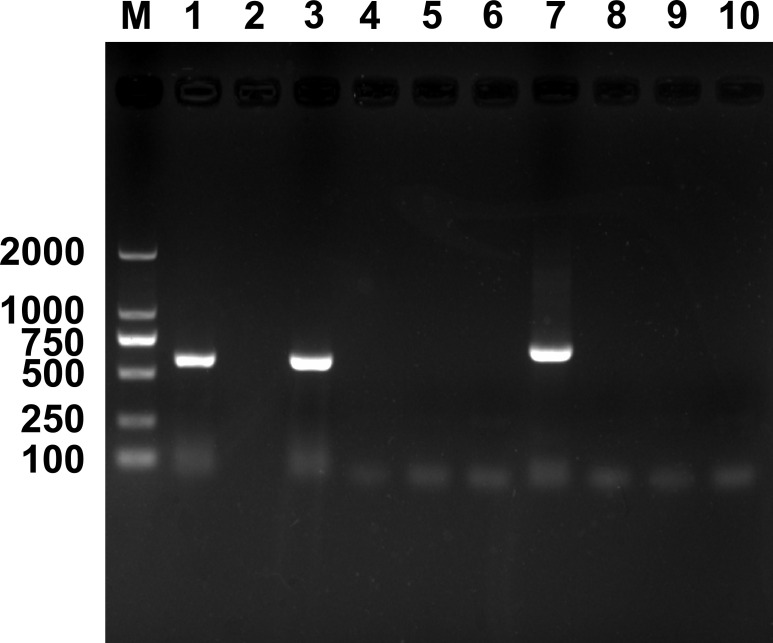
PCR detection of endosymbionts in *Encarsia formosa* that emerged from *Rickettsia*-negative *B tabaci* MEAM1 nymphs. M, DNA marker, from top 2,000, 1,000, 750, 500, 250, and 100 bp; lanes 1–10 are positive control (*Wolbachia*, *wsp* gene), negative control (ddH_2_O), *Wolbachia*, *Rickettsia*, *Cardinium*, *Hamiltonella*, *Hemipteriphilus*, *Arsenophonus*, *Fritschea*, and *Portiera*.

Results of PCR detection revealed that two endosymbionts, *Hemipteriphilus* (*16S rRNA* gene) and *Wolbachia* (*wsp* gene), were present in the parasitoid *E. formosa* (**Figure 1**), and their infection prevalence frequencies were 100% (90/90 in total). However, *Rickettsia* was absent in *E. formosa* (0/90 in total) (**Figure S1**).

### 3.2 *Rickettsia* transmission from *R* ^+^ whiteflies to parasitoids

**Figure 2:**
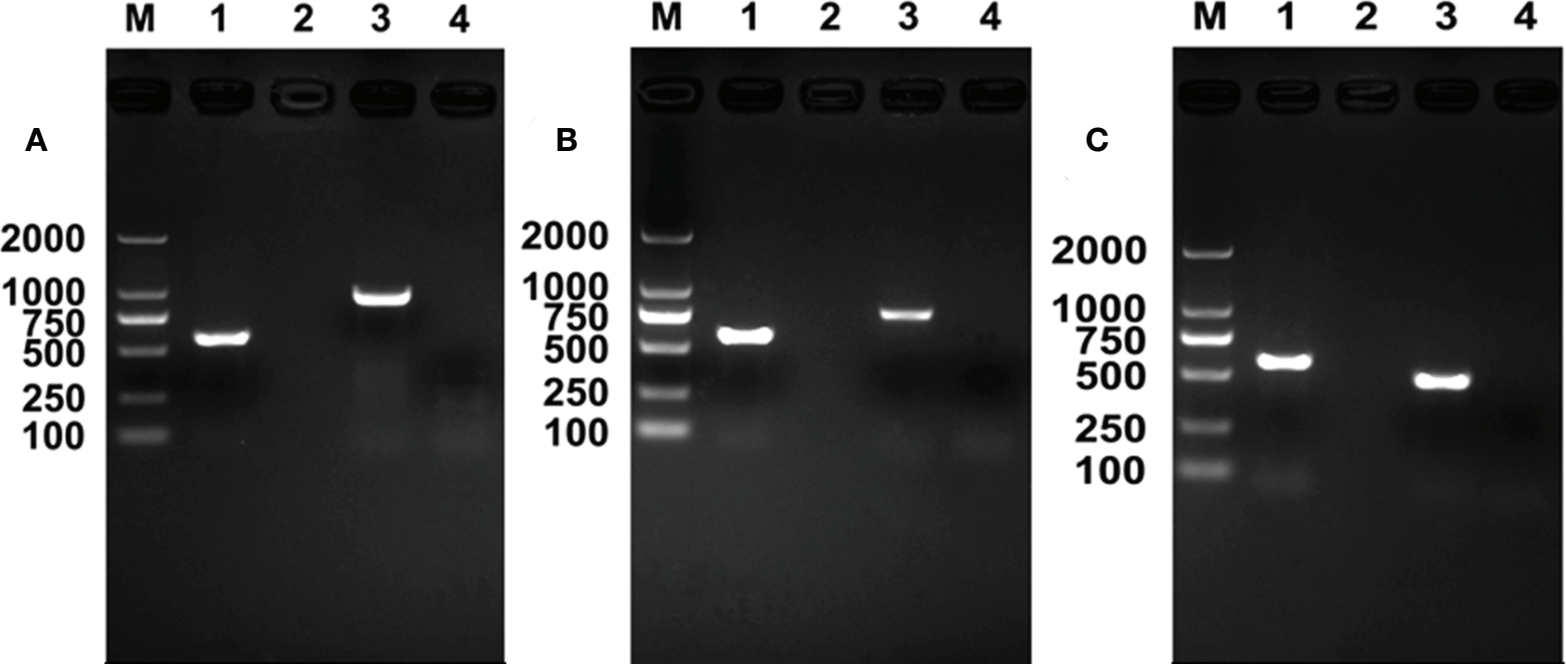
*Rickettsia* PCR detection in *Encarsia formosa* adults. M, DNA marker, from top 2,000, 1,000, 750, 500, 250, and 100 bp; lane 1, positive control (*Wolbachia*, *wsp* gene); lane 2, negative control (ddH_2_O); lane 3, *E formosa* that emerged from *Rickettsia*-positive populations; lane 4, *E formosa* that emerged from *Rickettsia*-negative populations. **(A)** *Rickettsia 16S rRNA* gene; **(B)** *Rickettsia gltA* gene; **(C)** *Rickettsia Pgt* gene.

Using *16S rRNA* gene, *gltA* gene, and *Pgt* gene, results of *Rickettsia* PCR detection revealed that, during the development of *E. formosa* in *R^+^* MEAM1 nymphs, *Rickettsia* was successfully transmitted from the whitefly host into the parasitoid (**Figure 2**).

### 3.3 Quantitative and FISH detection of *Rickettsia* in parasitoids

**Figure 3:**
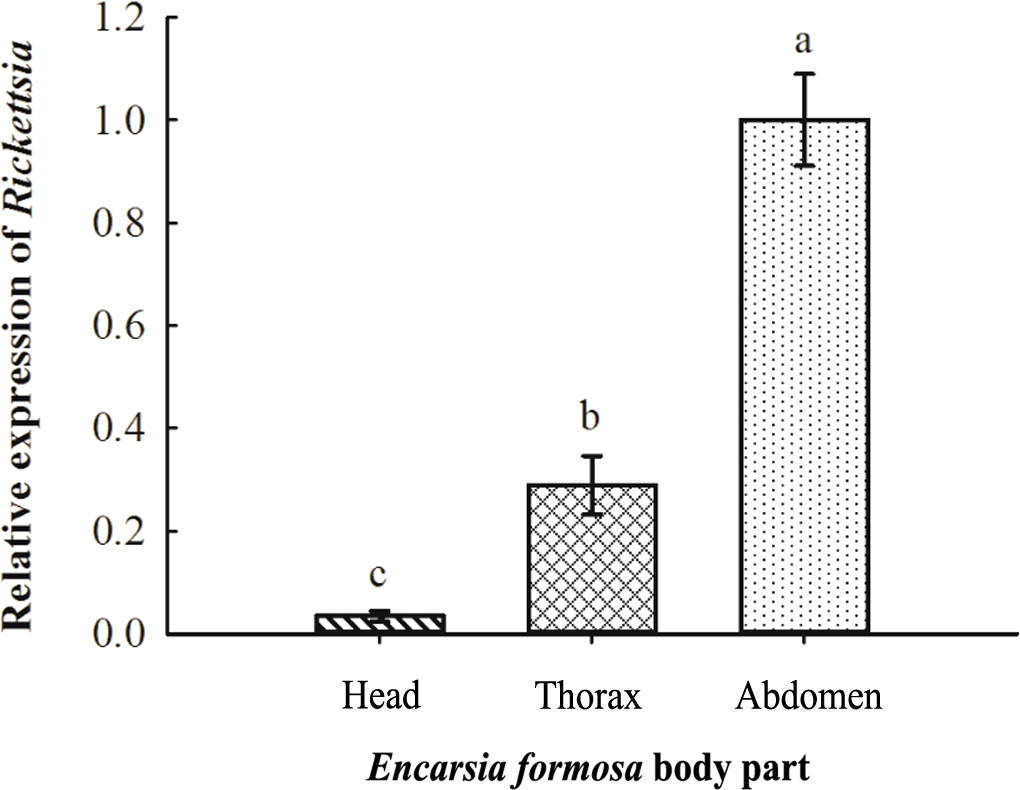
Acquisition of *Rickettsia* in the different tissues of *Encarsia formosa*. The column and error bars represent the fold change (titer) in mean ± SE (*n* = 3). The different letters above the bars indicate significant differences between different parts according to Duncan’s test (one-way ANOVA analysis, *p* < 0.01).

**Figure 4:**
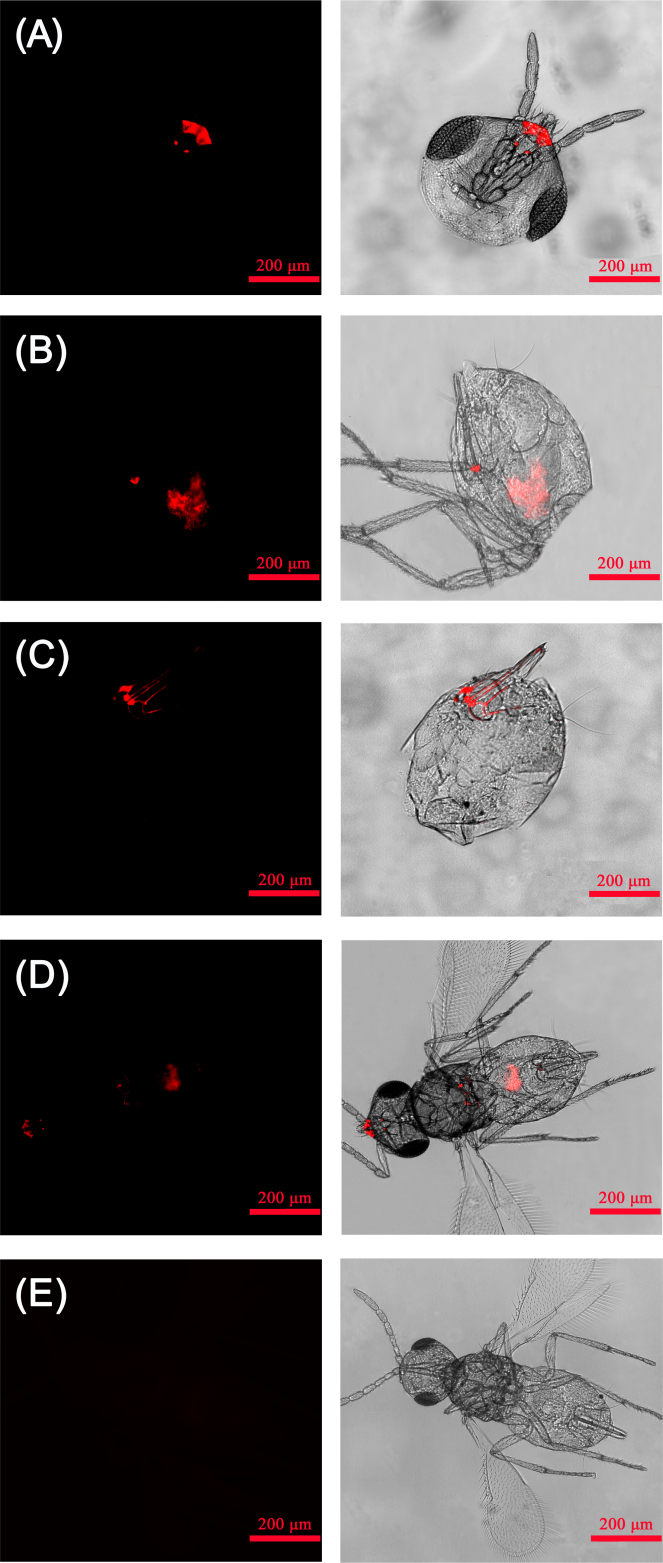
Localization of *Rickettsia* in adult *Encarsia formosa* parasitoids. **(A)** *Rickettsia* localized in the parasitoid mouthparts; **(B)** *Rickettsia* localized in the parasitoid digestive tract; **(C)** *Rickettsia* localized in the parasitoid ovipositors; **(D)** *Rickettsia* localized in the parasitoid head, thorax, and abdomen; **(E)** negative control-*E*. *formosa* parasitoid developed from *Rickettsia*-negative whitefly host. Left panels: fluorescence in dark field; right panels: fluorescence in bright field.

The relative titers of *Rickettsia* in different tissues of *E. formosa* were examined by using qPCR. Results showed that the parasitoid’s head, thorax, and abdomen were all infected with *Rickettsia*. Meanwhile, the titer of *Rickettsia* was highest in the abdomen (*F*_2, 6_ = 18.563, *p* < 0.01) (**Figure 3**). Results of FISH revealed that *Rickettsia* was mainly distributed in the mouthparts at the head, the ovipositor at the abdomen, the digestive tract of the abdomen, and the thorax. No specific FISH signal was observed in the head, thorax, and abdomen of negative control, i.e., the *E*. *formosa* individuals developed from *R^−^* whitefly hosts (**Figure 4**).

### 3.4 Persistence of *Rickettsia* in parasitoids

**Figure 5:**
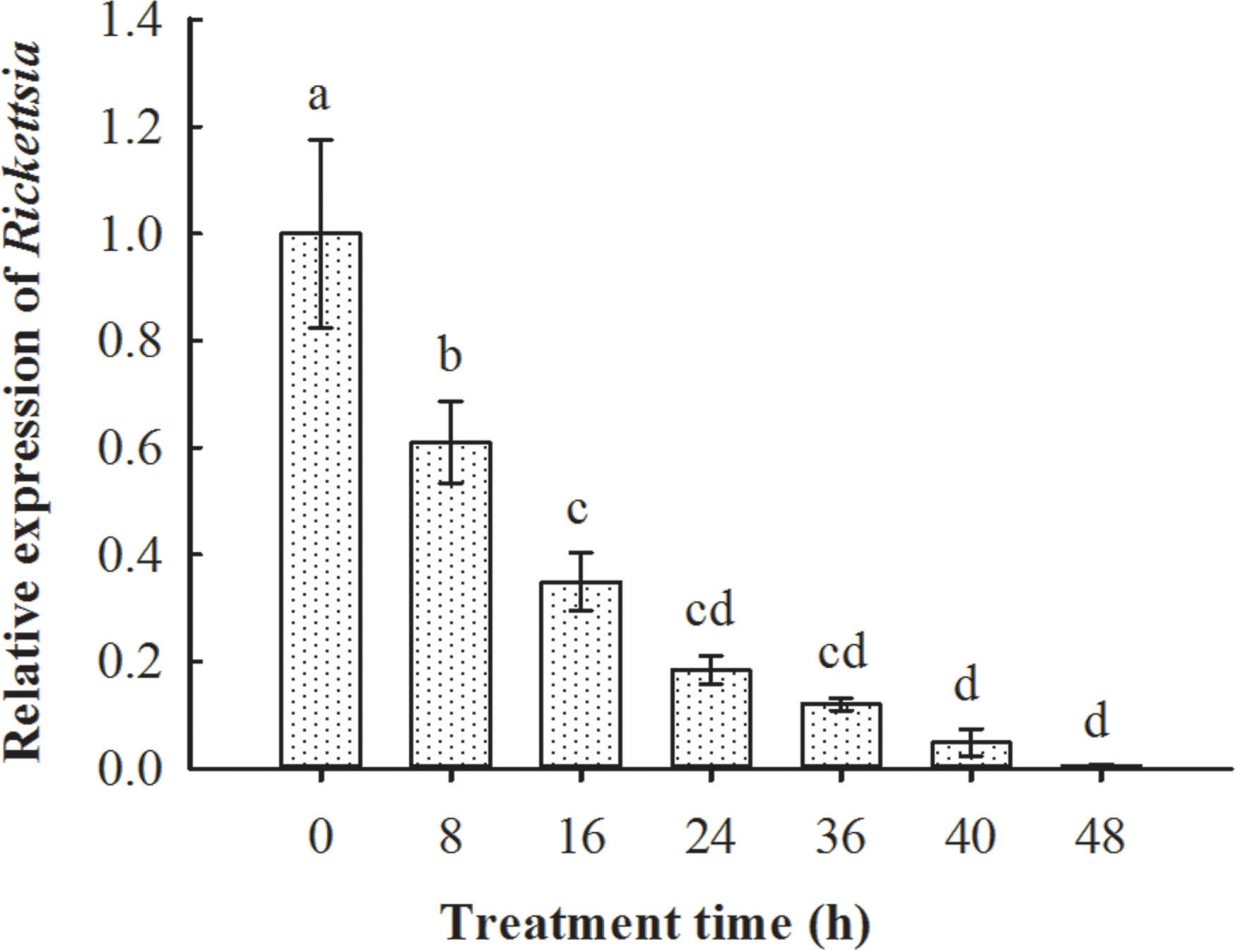
Persistence of *Rickettsia* in *Encarsia formosa* parasitoids that emerged from *Rickettsia*-positive *B tabaci* MEAM1 nymphs. The column and error bars represent the fold change (titer) in mean ± SE (*n* = 3). The different letters above the bars indicate significant differences between different treatment times according to Duncan’s test (one-way ANOVA analysis, *p* < 0.01).

The qPCR results suggested that, after the emergence of parasitoid adults from whitefly hosts, they are infected with *Rickettsia*. *Rickettsia* was retained in the following 48 h, although the titer was gradually reduced (*F*_6, 14_ = 22.103, *p* < 0.01; **Figure 5**).

### 3.5 *Rickettsia* transmission from parasitoids to *R^−^* whiteflies and its vertical transmission

**Figure 6:**
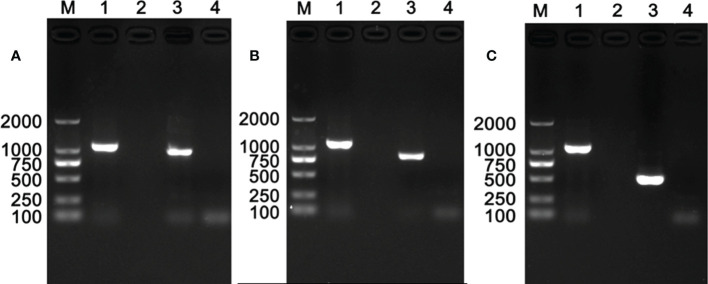
*Rickettsia* detection in recipient negative *B tabaci* MEAM1 nymphs. M, DNA marker, from top 2,000, 1,000, 750, 500, 250, and 100 bp; lane 1, positive control (*Portiera*, *16S rRNA* gene); lane 2, negative control (ddH_2_O); lane 3, surviving recipient *Rickettsia*-negative populations by parasitoids’ non-lethal probing and feeding; lane 4, *Rickettsia*-negative populations. **(A)** *Rickettsia 16S rRNA* gene; **(B)** *Rickettsia gltA* gene; **(C)** *Rickettsia Pgt* gene.

**Figure 7:**
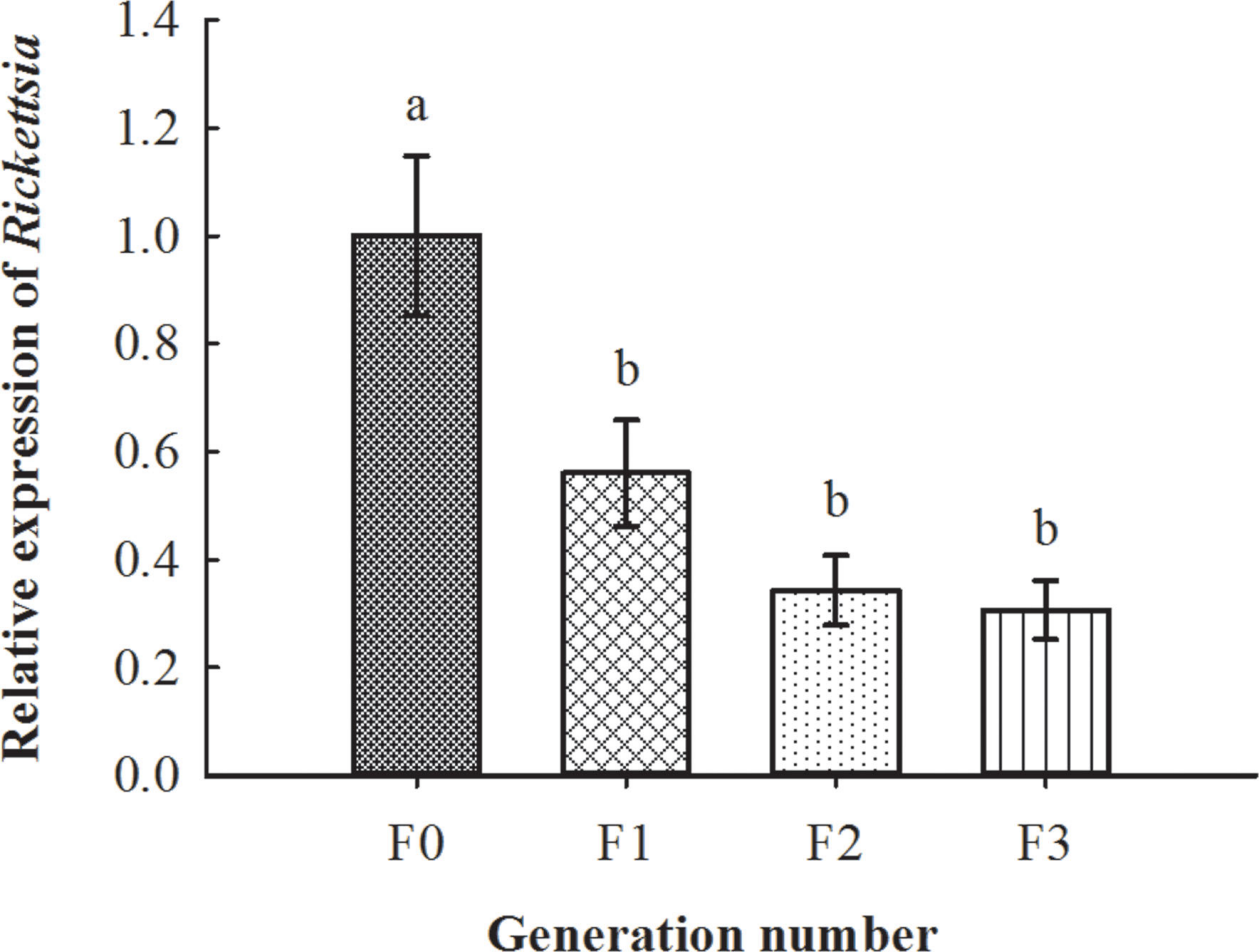
Vertical transmission situation of *Rickettsia* in recipient negative *B tabaci* MEAM1 nymphs. The column and error bars represent the fold change (titer) in mean ± SE (*n* = 3). The different letters above the bars indicate significant differences between different generations according to Duncan’s test (one-way ANOVA analysis, *p* < 0.01).

After the recipient MEAM1 nymphs (*R^−^*) were probed non-lethally and fed on by donor *E. formosa* (*R* ^+^) (i.e., vector parasitoids that previously developed from *R* ^+^ MEAM1 nymphs), PCR results demonstrated that the survived whitefly adults were infected with *Rickettsia* (**Figure 6**). Further qPCR detections showed that *Rickettsia* could vertically transmit up to F3 progenies of the recipient *R^−^* whiteflies (*F*_3, 8_ = 9.642, *p* < 0.01; **Figure 7**).

### 3.6 Phylogenetic analysis of *Rickettsia* in whiteflies and parasitoids

**Figure 8:**
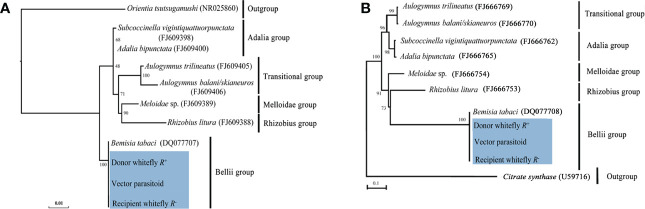
Phylogenetic analysis of *Rickettsia* in different *B tabaci* MEAM1 populations and *Encarsia formosa* parasitoids. **(A)** *16S rRNA* gene: *Orientia tsutsugamushi* was used as an outgroup; **(B)** *gltA* gene: *Citrate synthase* was used as an outgroup. The phylogenetic tree was constructed and analyzed by maximum likelihood (ML) method using 1,000 bootstrap replicates. Numbers at the nodes indicate the percentages of reliability of each branch of the tree. Branch length is drawn proportional to the estimated sequence divergence. Blue shadow part showed the different MEAM1 populations and parasitoids. “Donor whitefly *R* ^+^” represented donor *Rickettsia*-positive MEAM1; “Vector parasitoid” represented *Encarsia formosa*; “Recipient whitefly *R^−^*” represented recipient *Rickettsia*-negative MEAM1. They were all clustered into one branch within the *Bellii* group.

Results of the phylogenetic analysis revealed that horizontal transmission of *Rickettsia* in our study had high fidelity (**Figure S2**) between the *Rickettsia* donor and recipient populations of MEAM1 and the vector parasitoid *E. formosa*, and all the *Rickettsia* were clustered into one branch belonging to the *Bellii* group based on their *16S rRNA* and *gltA* gene sequences (**Figure 8**).

## 4 Discussion

There is growing evidence for the inter- and intra-specific horizontal transmission of endosymbionts among arthropod species revealing taxonomically far species carrying identical strains of endosymbionts (Ahmed et al., 2010; Zhang et al., 2013; Qi et al., 2019; Karut et al., 2020; Ren et al., 2020). Furthermore, transmission *via* parasitoids or other ecological interactions has long been proposed to mediate the horizontal transfer of endosymbionts from one species to another (Ahmed et al., 2015; Li et al., 2017a; Li et al., 2017b).

*E. formosa* is a dominant endoparasitoid of whitefly, and its endosymbionts have been widely studied (Zchori-Fein et al., 2001; Li et al., 2011; Xue et al., 2017). Previous studies have reported that the prevalence of the endosymbionts in insect hosts may vary, because it correlates with the host’s development, diet, temperature, and other abiotic factors (Toju and Fukatsu, 2011). For example, Fan (2013) found that *E. formosa* was infected with *Wolbachia* and the infection rate was 100%; Xue et al. (2017) revealed *Rickettsia* infection in *E. formosa* and *Encarsia sophia*, but infections of *Wolbachia* and *Hamiltonella* were only detected in *E. formosa*. In our current study, the infection status of *Rickettisa* differed within *B. tabaci* MEAM1 hosts; *E. formosa* wasps were *Rickettsia* infected when they were developed from *R^+^* MEAM1 nymphs, while they were *Rickettsia* uninfected when they were developed from *R^−^* MEAM1 nymphs.

Our study reported transmission of endosymbionts between two trophic levels by demonstrating the efficient phoretic transfer of *Rickettsia* through the parasitoid *E. formosa* between infected and uninfected individuals of whitefly *B. tabaci* MEAM1. Before this study, parasitoids had been revealed to be able to vector the horizontal transmission of endosymbionts inter- and intra-specifically (Heath et al., 1999; Chiel et al., 2009; Ahmed et al., 2015). For instance, Heath et al. (1999) found that *Wolbachia* can be transmitted from an infected host, *Drosophila simulans*, to an attacking endoparasitoid, *Leptopilina boulardi*, and subsequently undergo diminishing vertical transmission in this new host population. Chiel et al. (2009) reported that adults of three parasitoid species, *Eretmocerus emiratus*, *Eretmocerus eremicus*, and *Encarsia pergandiella*, frequently acquired *Rickettsia via* contact with infected whiteflies, but the rate of infection declined sharply within a few days of wasps being removed from infected whiteflies. Our results are similar to the finding in a previous study by Ahmed et al. (2015) that the bacterial endosymbiont, *Wolbachia*, could be detected in the mouthparts and ovipositors of *E. furuhashii*.

Horizontal transmission of endosymbionts between hosts and parasitoids is mainly unidirectional, from the hosts to the parasitoids. However, it has been suggested that horizontal transmission from parasitoids to their hosts would be unlikely as parasitized hosts die. The mode of transmission we have described here relies on the fact that parasitoids do not always kill hosts with which they interact (Gehrer and Vorburger, 2012; Ahmed et al., 2015). As previously reported, two parasitoids, *Lysiphlebus fabarum* and *Aphidius colemani*, can transfer *H. defensa* and *R. insecticola* by sequentially stabbing infected and uninfected individuals of their host, *Aphis fabae*, then establishing new, heritable infections (Gehrer and Vorburger, 2012). Our previous study revealed that non-lethal probing of uninfected *B. tabaci* AsiaII7 nymphs by parasitoids carrying *Wolbachia* resulted in new stable infected *B. tabaci* individuals (Ahmed et al., 2015). In addition, our current study reported that *Rickettsia* was detected in the recipient *R^−^* MEAM1 adults, and the vertical transmission can occur up to F3 generations. Molecular phylogenetic analysis of *Rickettsia* showed 100% fidelity in donor *R* ^+^ MEAM1, vector *E. formosa* parasitoids, and recipient *R^−^* MEAM1, contrary to other studies, in which bacterial endosymbionts transmit horizontally with poor fidelity (Kang et al., 2003; Kikuchi and Fukatsu, 2003; Riegler et al., 2004) or failed to persist after horizontal transmisison (Chiel et al., 2009). On the other hand, our current study suggested that, when releasing *E. formosa* parasitoids to manage whitefly pests, the *Rickettsia* infection status of whitlefly should be determined. This is because *E. formosa* parasitoids, which have probed or fed upon the *R^+^* MEAM1 nymphs, could change the biology of recipient *R^−^* MEAM1 nymphs and might enhance its pestiferous nature (Oliver et al., 2003; Himler et al., 2011).

The ecological dynamics of endosymbionts in their inter- and intra-specific horizontal transmission and their interactions with insect hosts and their parasitoids could be the focus of future research (Karut et al., 2020; Bao et al., 2021). In this study, we provided a novel evidence for the parasitoid-vectored horizontal transmission of bacterial endosymbiont *Rickettsia* between different whitefly hosts. Our current study will help understand why the endosymbionts are so ubiquitous in arthropod communities and why phylogenetically distinct arthropods often harbor closely related endosymbionts in nature (Ahmed et al., 2013; Ahmed et al., 2015; Weinert et al., 2015).

**Figure 9:**
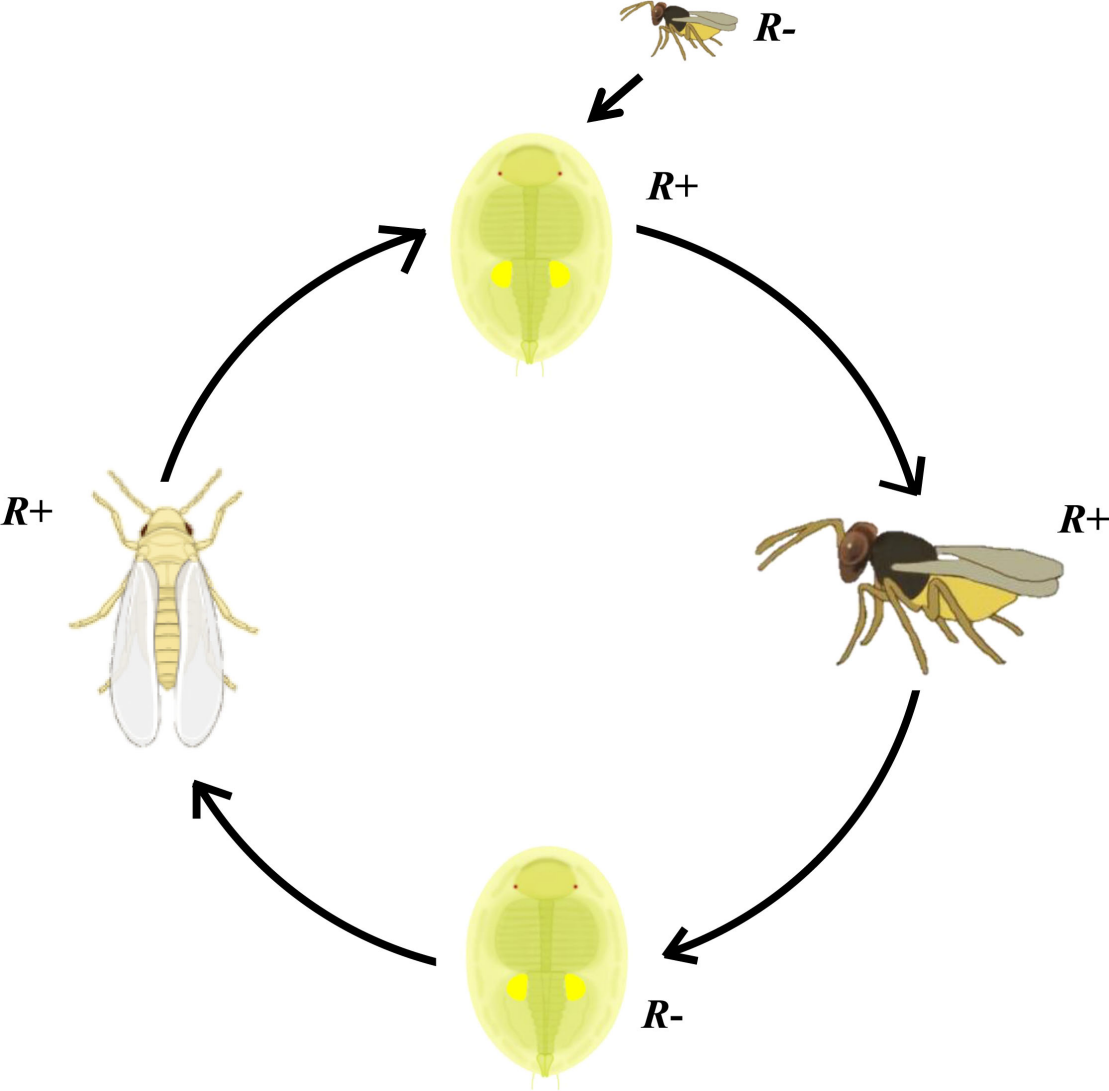
Schematic overview of *Rickettsia* transmission vectored by *Encarsia formosa* parasitoid. Parasitoid *Encarsia formosa* can acquire *Rickettsia* from these *Rickettsia*-positive (*R* ^+^) MEAM1 nymphs and carry it in their ovipositors. After probe checking *Rickettsia*-negative (*R^−^*) MEAM1 nymphs with ovipositor, if parasitized MEAM1 nymphs survive from the parasitizing check, *Rickettsia* can spread into the MEAM1 adults and remain at least up to F3 generation.

In conclusion, our current study reveals that *Rickettsia* endosymbionts can be picked up by the parasitoid *E. formosa* during their development in *Rickettisia*-infected (*R* ^+^) MEAM1 nymphs and that it can persist in the parasitoid adult for at least 48 h following wasp emergence. During its persistence in the parasitoid, random non-lethal probing of *Rickettisia* uninfected (*R^−^*) MEAM1 nymphs by these *Rickettsia-*carrying *E. formosa* resulted in newly infected MEAM1 individuals (**Figure 9**). These findings may help to explain why *Rickettsia* is so abundant in arthropods and may have significant implications during parasitoid-based biological control of whitefly and for understanding the multifaceted interactions between endosymbionts, insects, and parasitoids.

## Data availability statement

The original contributions presented in the study are included in the article/supplementary material. Further inquiries can be directed to the corresponding author.

## Author contributions

This article was originally designed by YL, NM, MZA, and B-LQ; YL, Z-QH, QW, JP, and Y-TZ carried out experiments; NM, CLM, and MZA also help to analyze the phenotype as well as the data; YL also participated in data analysis; YL, MZA and B-LQ wrote the paper. All authors gave final approval for publication.

## Supplementary material

Supplementary Figure 1 PCR detection of three endosymbionts, *Hemipteriphilus*, *Wolbachia*, and *Rickettsia* in *Encarsia formosa* parasitoids, related to . M, DNA marker, from top 2000, 1000, 750, 500, 250, 100bp; lane 1-90, 90 *Encarsia formosa* parasitoids. **(A)** *Hemipteriphilus* **; (B)** *Wolbachia*; **(C)** *Rickettsia.*

Supplementary Figure 2 The sequence alignment of *16S rRNA* and *gltA* genes of *Rickettsia* from donor, recipient *B*. *tabaci* MEAM1 and *Encarsia formosa* parasitoids, related to **(A)** *16S rRNA* gene; **(B)** *gltA* gene.
