# Supplementary figures and images for: Parasitoid-mediated horizontal transmission of Rickettsia between whiteflies
Source: Front Cell Infect Microbiol. 2023 Jan 4;12:1077494. doi: 10.3389/fcimb.2022.1077494 (PMC9846228; doi:10.3389/fcimb.2022.1077494)

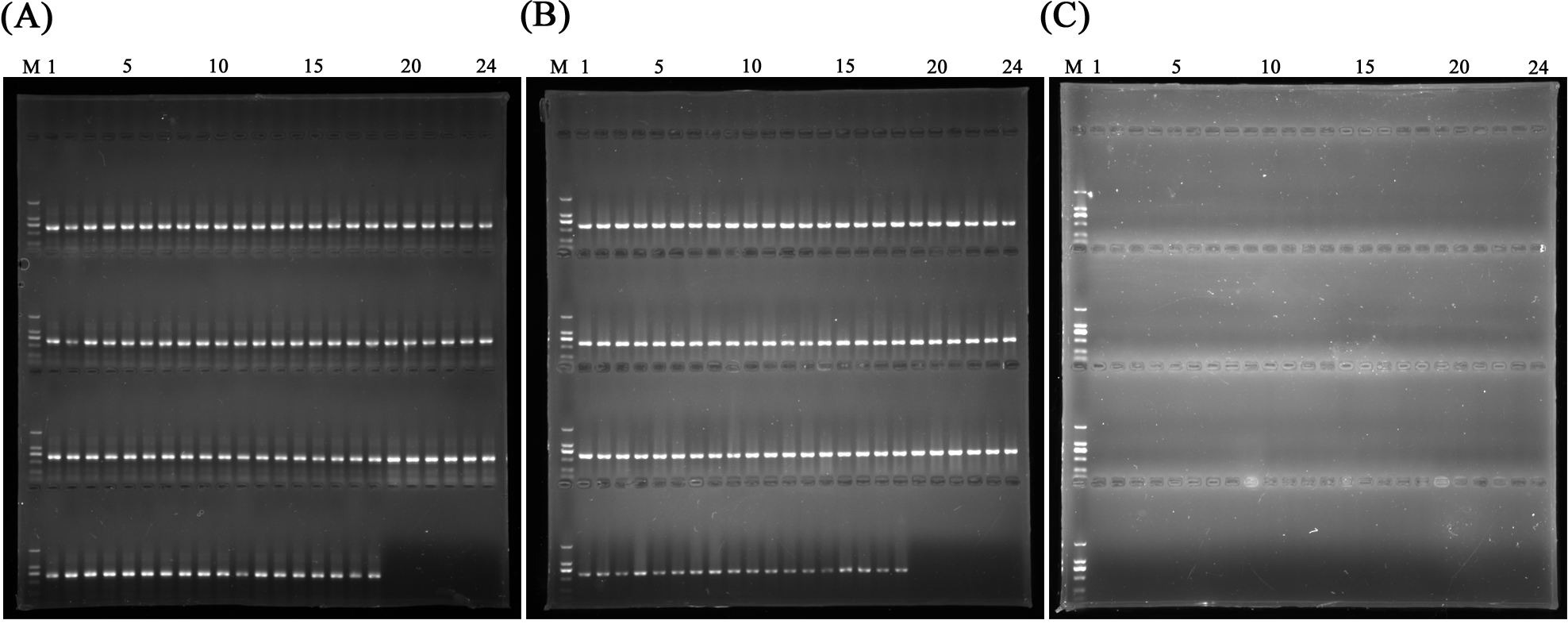

Supplement: Supplementary Figure 1 — PCR detection of three endosymbionts, Hemipteriphilus, Wolbachia, and Rickettsia in Encarsia formosa parasitoids, related to . M, DNA marker, from top 2000, 1000, 750, 500, 250, 100bp; lane 1-90, 90 Encarsia formosa parasitoids. (A) Hemipteriphilus ; (B) Wolbachia; (C) Rickettsia. [file DataSheet_1.zip › Supplemental file/Figure S1.tif]

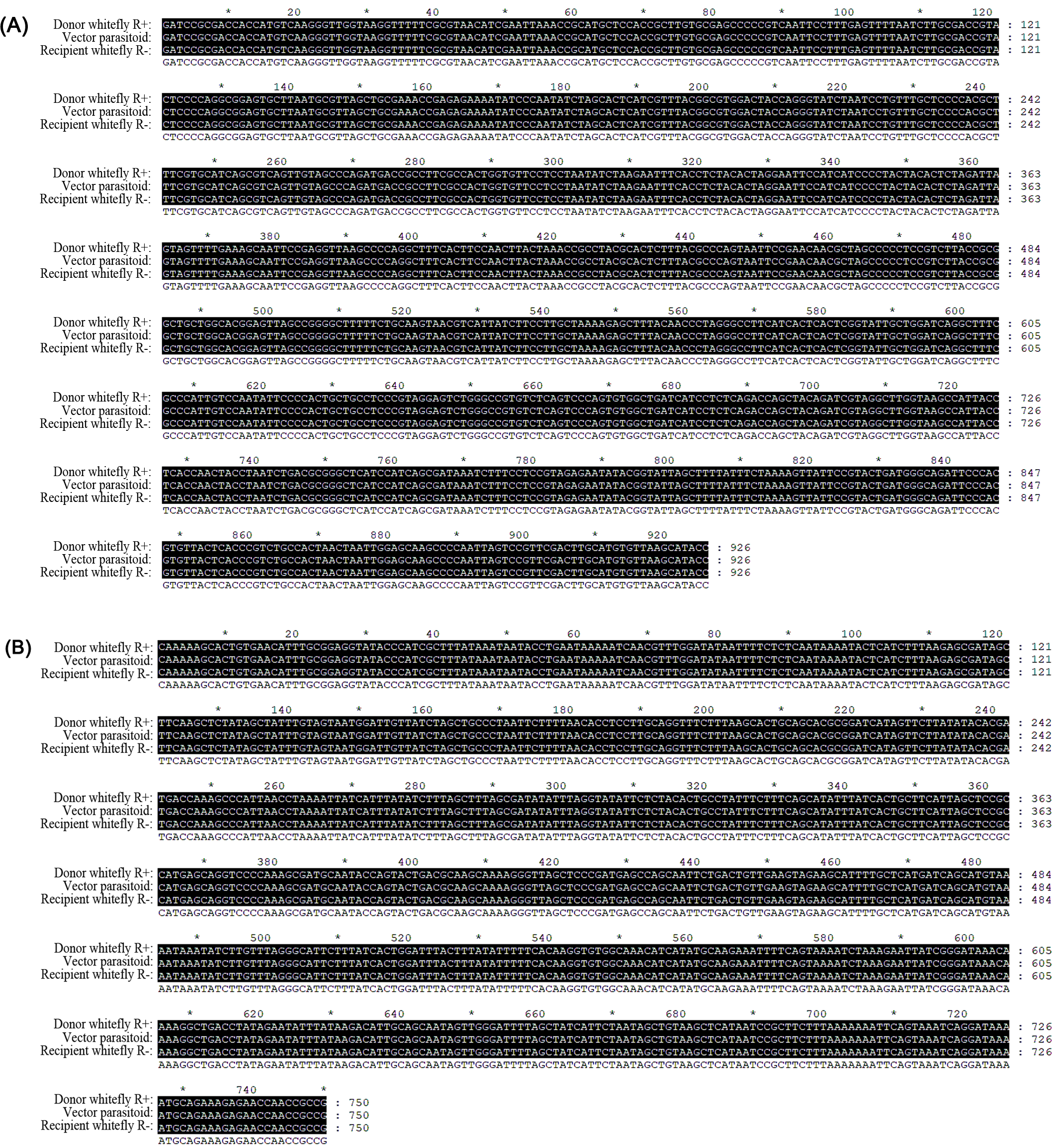

Supplement: Supplementary Figure 1 — PCR detection of three endosymbionts, Hemipteriphilus, Wolbachia, and Rickettsia in Encarsia formosa parasitoids, related to . M, DNA marker, from top 2000, 1000, 750, 500, 250, 100bp; lane 1-90, 90 Encarsia formosa parasitoids. (A) Hemipteriphilus ; (B) Wolbachia; (C) Rickettsia. [file DataSheet_1.zip › Supplemental file/Figure S2.tif]
